# Real-world tafamidis experience in hereditary transthyretin amyloidosis with peripheral neuropathy in Brazil

**DOI:** 10.1055/s-0044-1793936

**Published:** 2025-01-15

**Authors:** Luiz Felipe Pinto, Marcus V. Pinto, Paula Accioli, Gabriela Amorim, Renata Gervais de Santa Rosa, Moises Dias, Mariana Guedes, Carlos P. Gomez, Roberto C. Pedrosa, Marcia Waddington-Cruz

**Affiliations:** 1 Universidade Federal do Rio de Janeiro, Hospital Universitário Clementino Fraga Filho, Centro de Estudos em Paramiloidose Antônio Rodrigues de Mello, Rio de Janeiro RJ, Brazil.; 2 Mayo Clinic, Department of Neurology, Rochester MN, United States.

**Keywords:** Polyneuropathies, Prealbumin, Amyloid Neuropathies, Familial, Amyloidosis, Familial

## Abstract

**Background** Tafamidis is a kinetic stabilizer that binds to the transthyretin (TTR) gene, inhibiting its dissociation. It is the only disease-modifying treatment for hereditary TTR amyloidosis with peripheral neuropathy (ATTRv-PN) available in the National Therapeutic Form (Formulário Terapêutico Nacional, FTN, in Portuguese) of the Brazilian Unified Health System (Sistema Único de Saúde, SUS, in Portuguese).

**Objective** To assess if the efficacy and safety of tafamidis in the Brazilian real-world experience are comparable to the results of clinical trials.

**Methods** We retrospectively studied all patients with ATTRv-PN evaluated at our center from September 2011 to March 2022 (data cut-off) who were initiated on tafamidis and had at least 1 follow up visit 6 months after the initiation of the drug treatment. Neurologic and functional outcomes were compared from day 1 (D1) of the tafamidis treatment (baseline) to the last follow-up.

**Results** In total, 33 patients were included, 18 (55%) of whom were female. All patients were carriers of the V30M mutation of ATTRv-PN, and 20 (61%) presented early onset (EO) ATTRv-PN. At baseline, the median age of the sample was of 40 (interquartile range [IQR]: 36–68) years, the median Neuropathy Impairment Score (NIS) was of 10 (6–24) points, and the median body mass index (BMI) was of 26 (23–28) kg/m ^2^ . The mean follow-up time was of 3.4 years. At the last follow-up, the BMI, the neurological impairment, and the level of disability slightly worsened compared with baseline, while the findings of the nerve conduction studies remained stable. These same results were observed across EO and late-onset (LO) ATTRv-PN patients. A total of 25 (75.8%) patients were considered responders, and 8 (24.2%), non-responders.

**Conclusion** The efficacy and safety of tafamidis reported in clinical trials is expandable to the Brazilian real-world scenario in EO and late-onset (LO) ATTRv-PN.

## INTRODUCTION

Hereditary transthyretin amyloidosis (ATTRv) is an autosomal dominant disorder caused by mutations in the *transthyretin* ( *TTR* ) gene, which is a tetrameric transport protein produced mainly by the liver that carries retinol and thyroxin throughout the body. The mutation provokes an amino acid exchange, ensuing protein misfolding and tetramer dissociation. The deposition of monomers will cause amyloid deposits mainly in the nerves, heart, and kidney. 1

A severe, progressive, and disabling disease, ATTRv presents variable phenotypes depending on the mutation, but it is classically divided into the polyneuropathy and cardiomyopathy forms. The most common mutation in Brazil is V30M (p.Val50Met), which causes a peripheral neuropathy-predominant phenotype (ATTRv-PN). 2 In the United States, the most common variant is Val122ile (p.Val142Ile), which leads to a cardiomyopathy-predominant phenotype (ATTRv-CM) and is present in 3.5% of African Americans. 3 Once TTR amyloid is formed, it cannot be cleared with the therapy currently available. Therefore, establishing an early diagnosis and treatment are paramount.

For many years, liver transplantation (LT) was the only available treatment for ATTRv-PN; however, nowadays, it is rarely recommended. Additionally, the costs associated with LT are high, and the procedure carries significant morbidity and mortality. Over the past 20 years, many new medications have been developed which are much safer than LT. There are two main categories of drugs: the tetramer stabilizers and the gene silencers. The stabilizers were the first to be developed, and they act by directly binding to the tetramers with high affinity and preventing the monomers from dissociating. The gene silencers act in the *TTR* mRNA, preventing it from being translated. 3

Tafamidis is a kinetic stabilizer that binds to TTR with high affinity, inhibiting its dissociation. It was the first drug approved for the treatment of ATTRv-PN in Europe, South America, and Asia, and it acts by slowing down the progression of the disease, but does not completely halt it. 4 It is very safe and has shown for more than10 years its efficacy and very low rate of adverse events. 5 The drug has not been approved yet for ATTRv-PN by the US Food and Drug Administration (FDA), although it has been recently approved to treat ATTRv cardiomyopathy. Tafamidis was approved by the Brazilian Health Regulatory Agency (Agência Nacional de Vigilância Sanitária, ANVISA) in 2017 and included in the National Therapeutic Form (Formulário Terapêutico Nacional, FTN) of the Brazilian Unified Health System (Sistema Único de Saúde, SUS) in 2018. We herein describe a 10-year-long real-world experience with tafamidis in the Brazilian National Amyloid Referral Center (Centro de Estudos em Paramiloidose Antônio Rodrigues de Mello, CEPARM).

## METHODS

We retrospectively studied all patients with ATTRv-PN evaluated at our center, from September 2011 to March 2022 (data cut-off), who were initiated on daily doses of 20 mg of tafamidi and had at least 1 follow up visit 6-months after baseline. All patients signed an informed consent form, and the study was approved by the Review Board at Universidade Federal do Rio de Janeiro. The patients were assessed by a neurologist, a nephrologist, and a cardiologist at each visit, as per our center's protocol. All patients were carriers of a known pathogenic variant in the *TTR* gene and symptomatic ATTRv-PN.

To be included in the present study, the patients had to present signs of peripheral neuropathy on the neurologic examination or nerve conduction studies or SUDOSCAN (Impeto Medical, Issy-les-Moulineaux, France) test, or positive tissue biopsy. The following assessments were performed on each visit: Polyneuropathy Disability Score (PND); Neuropathy Impairment Score (NIS); Karnofsky Performance Status (KPS); body mass index (BMI); electrocardiogram (ECG); transthoracic echocardiogram; and conduction studies of the right peroneal motor and sural nerves.

The patients were divided into the late-onset (LO) ATTRv-PN group, with age at symptom onset ≥ 50 years, and the early-onset (EO) ATTRvPN) group, with age at symptom onset < 50 years. We classified them as tafamidis non-responders if they presented an increase on the NIS of 7 or more points per year since the start of the treatment (tafamidis day 1, D1). 6 7 Interventricular septum hypertrophy was defined as > 12 mm (ATTRv-CM). 3

### Statistical analysis

The categorical variables were expressed as numbers and percentages, and the differences between the groups were compared using the Fisher's Chi-squared (χ ^2^ ) exact test. The continuous variables were expressed as median and interquartile range (IQR) values, and the comparison between the groups was performed through Kruskal-Wallis test. Pairs matched by baseline and the last follow-up matched were tested with the Wilcoxon-rank signed test. The JMP (SAS institute, Cary, NC, USA) software, version 17, was used for all statistical analysis. All the tests were two-sided, and *p* -values < 0.05 were considered significant.

## RESULTS

Of the 45 patients who started the treatment with tafamidis during the study period, 33 were included in the present study. All patients were carriers of the V30M mutation of ATTRv-PN. The sample was composed of 18 (55%) female patients, and the EO-ATTRv-PN group contained 20 (61%) subjects. The median age at baseline was of 40 (IQR: 36–68) years ( Table 1 ). The Median baseline NIS was of 10 (IQR: 6–24) points, and the median BMI, of 26 (23–28) kg/m ^2^ . In total, 17 (59%) patients had an abnormal ECG and 3 presented ATTRv cardiomyopathy (all LO). The mean time from baseline to the last follow-up was of 3.4 years. Besides age at symptom onset, there were no significant differences in terms of demographic, neurologic or cardiac features between the EO- and LO-ATTRv-PN patients. A total of 3 patients had used inotersen previously (3–5 years before initiating tafamidis, from 2015 to 2019) and changed to tafamidis due to side effects from inotersen (thrombocytopenia: *n* = 2; and glomerulonephritis: *n* = 1).

**Table 1:** Demographic, clinical, and electrodiagnostic findings at tafamidis day 1 (baseline)

		Total	EO-V30M patients (N = 20)	LO-V30M patients (N = 13)	*p* -value
Female sex: n (%)		18 (55)	11(55)	7 (54)	1.0
Age (years): median (IQR)		40 (36–68)	36 (30–39)	65 (62–68)	< 0.0001
Disease duration (years): median (IQR)		3 (1–4)	2.5 (1–4)	3 (2.5–4.5)	0.3512
NIS: median (IQR)		10 (6–24)	8 (2–17)	14 (7–29)	0.1269
BMI: median (IQR)		26 (23–28)	25 (22–27)	26 (25–29)	0.1846
KPS: median (IQR)		90 (80–90)	90 (80–90)	90 (80–90)	0.4
PND: n (%)	I	27 (82)	17 (85)	10 (77)	0.5874
	II	5 (15)	3 (15)	2 (15)	
	IIIa	1 (3)	0	1 (8)	
Sural amplitude (µV): *n* = 29		2.9 (0–7)	3.6 (0–9.7)	0 (0–3.8)	0.0666
Peroneal amplitude (mV): *n* = 29		2.4 (0.5–4.8)	3.1 (0.7–5)	1.1 (0–4)	0.2142
Abnormal ECG:n (%) – *n* = 29		17 (59)	9 (53)	8 (67)	0.7032
Abnormal IVS:n (%) – *n* = 25		3 (8)	0	3 (30)	0.0522

Abbreviations: BMI, body mass index; ECG, electrocardiogram; EO, early-onset; IQR, interquartile range; IVS, interventricular septum; LO, late-onset; KPS, Karnofsky Performance Status; NIS, Neuropathy Impairment Score; PND, Polyneuropathy Disability Score.

**Figure 1:**
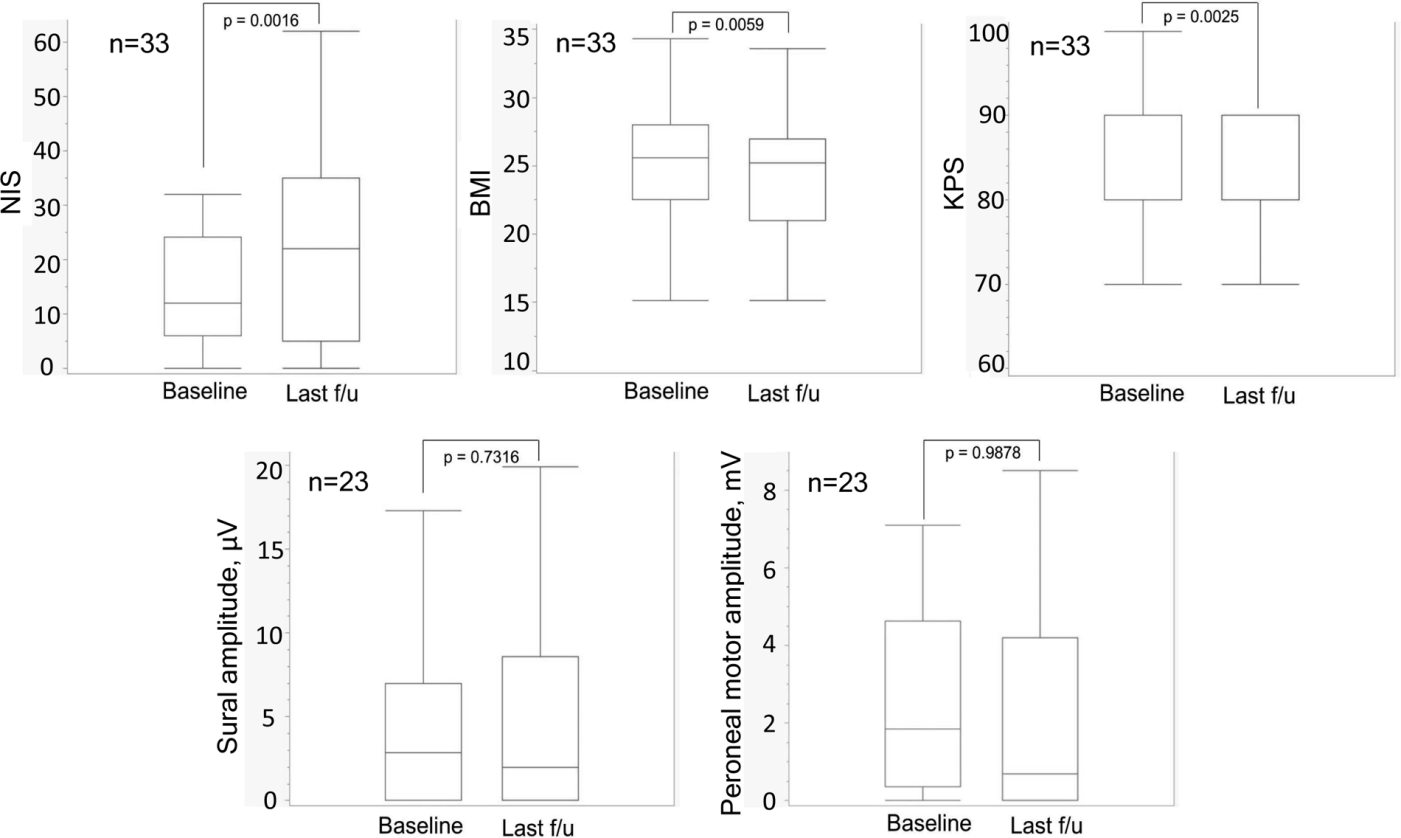
Abbreviations: NIS, Neuropathy Impairment Score; KPS, Karnofsky Performance Status; BMI, body mass index. Matched comparisons of neurological and functional outcomes from baseline (tafamidis day 1, D1) to the last follow-up.

**Figure 2:**
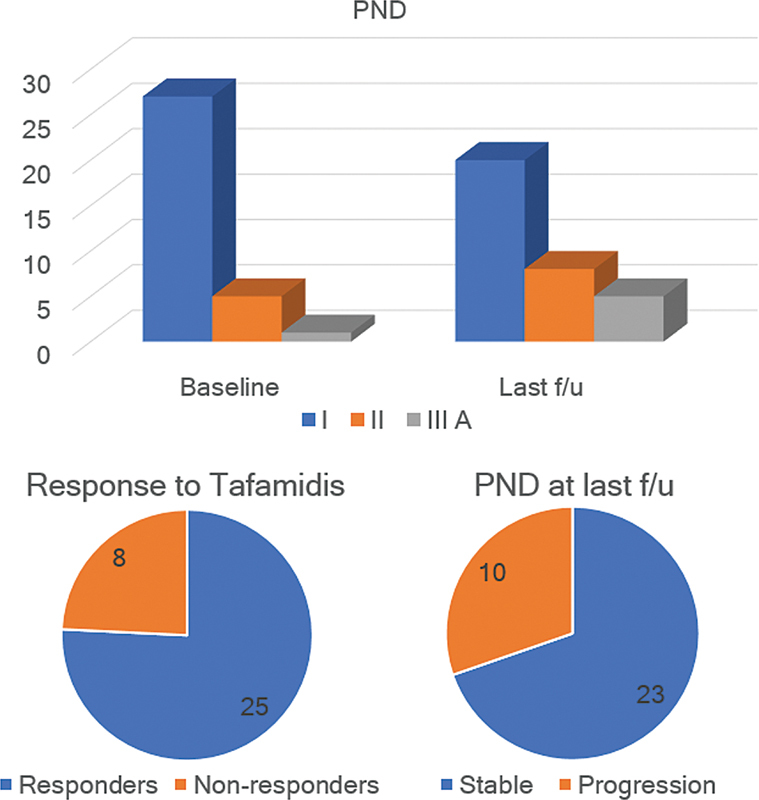
Abbreviation: PND, Polyneuropathy Disability Score. Progression of disability at the last follow-up and response to tafamidis.

At the last follow-up, the BMI, neurological impairment, and level of disability slightly worsened compared with baseline; however, the sural nerve's sensory amplitude, and the peroneal nerve's compound muscle action potential (CMAP) amplitude remained stable ( Figure 1 ). This same result was observed across EO- and LO-ATTRv-PN patients. Overall, 25 (75.8%) patients were considered responders, and 8 (24.2%), non-responders ( Figure 2 ). Similar results were observed among LO-ATTRv-PN patients ( *n* = 13; 10 responders and 3 nonresponders), patients who previously used inotersen ( *n* = 3; 2 responders and 1 nonresponder), and those who presented cardiomyopathy ( *n* = 3; 3 responders). The median rate of NIS progression was of 0 points per year in responders and of 11.3 per year in nonresponders. There were 10 nonresponders who worsened the PND stage in at least 1 point compared with baseline ( Figure 2 ). Furthermore, 8 outpatients discontinued tafamidis between 2019 and 2021 to join the Helios 8 and Neuro-Transform 9 trials. Comparing the baseline characteristics of responders and non-responders, there were no differences between the groups ( Table 2 ).

**Table 2:** Comparison of baseline characteristics of tafamidis in responders versus nonresponders

		Responders (N = 25)	Non-responders (N = 8)	*p* -value
Female sex: n (%)		15 (60)	3 (38)	0.4184
Age at tafamidis initiation (years)		41 (36–63)	36 (30–70)	0.8003
LO-V30M patients: n (%)		10 (40)	3 (38)	1.0
Disease duration (years): median (IQR)		3 (1–4)	3 (1–5)	0.8985
NIS: median (IQR)		10 (4–26)	15 (9–24)	0.3326
BMI: median (IQR)		26 (24–29)	26 (19–27)	0.3890
KPS: median (IQR)		90 (80–90)	85 (70–90)	0.6029
PND: n (%)	I	20 (80)	7 (88)	0.7234
	II	4 (16)	1 (12)	
	IIIa	1(4)		
Sural amplitude (µV): *n* = 29		3.5 (0–8.1)	1.2 (0–3.7)	0.3017
Peroneal amplitude (mV): *n* = 29		2.8 (0.5–5.3)	1(0.3–3.8)	0.3731
Abnormal ECG: n (%) – *n* = 29		14 (61)	3 (50)	0.6693
Abnormal IVS: n (%) – *n* = 25		3 (15)	0	1.0

Abbreviations: BMI, body mass index; ECG, electrocardiogram; IQR, interquartile range; IVS, interventricular septum; LO, late-onset; KPS, Karnofsky Performance Status; NIS, Neuropathy Impairment Score; PND, Polyneuropathy Disability Score.

No patient stopped tafamidis to undergo LT, neither were there deaths during the study period. No adverse event nor serious adverse events were considered related to tafamidis.

## DISCUSSION

The current study showed that the efficacy and safety of tafamidis reported in clinical trials is expandable to the Brazilian real-world scenario. Most patients benefited from the therapy, most remained in the same PND stage, there was no significant neurophysiological worsening of the peripheral neuropathy, and the KPS, BMI, and NIS only worsened slightly over a long period of time. Tafamidis is the only ATTRv disease-modifying therapy available in the SUS. The drugs inotersen and patisiran were recently approved by ANVISA, but the high costs make them a limited option to our ATTRv-PN population, as private health insurers do not cover these medications, and the SUS cannot afford them.

A tafamidis phase-II/-III randomized, double-blinded, placebo-controlled clinical trial (FX-005) showed a greater proportion of NIS-Lower Limbs (NIS-LL) responders among the tafamidis patients compared to those who received placebo (60.0 versus 38.1% respectively; *p* = 0.041) and better quality of life in the tafamidis group (Least-Square Norfolk Quality of Life-Diabetic Neuropathy total score: 8.8-point difference; *p* = 0.045) using efficacy-evaluable analysis. 10 This trial lasted for 18 months and was followed by an open label extension study (FX-006), which confirmed that the benefits were sustained for 30 months, and that early initiation of tafamidis was associated with better responses and outcomes. 11 Other studies 12 demonstrated that this drug caused a long-term (6 years) delay in neurological and nutritional status deterioration in V30M patients.

Real-world studies have shown mixed results regarding tafamidis efficacy around the globe. In 252 Portuguese subjects (V30M in 92.5% of the cases and mean age of 40.4 years), tafamidis significantly slowed disease progression during the 2 years of follow-up, when compared with nonrandomized untreated subjects. 13 Patients on tafamidis presented slower progression on the NIS-LL and its subscales, as well as lower levels of deterioration in the Norfolk Quality of Life scale. There were no significant differences in terms of BMI and KPS score between the groups. 13 In a large Portuguese natural history study, 5 patients with EO stage-I V30M ATTRv-PN on tafamidis presented approximately 91% of reduction in the mortality risk compared with untreated patients.

Monteiro et al. 14 retrospectively studied 210 patients with V30M ATTR with predominantly neuropathic phenotype treated with tafamidis for 18 to 66 months. The patients were classified by an expert as responders, partial responders, and non-responders. In total, 72 patients were classified as responders (34%; no disease progression, NIS change from baseline ≤ 0). Non-responders (61; 29%) and partial responders (76; 36%) presented worsening of sensory, motor and autonomic neuropathy (non-responders: average NIS increase of 5.9/year; partial responders: 1.8/year). Milder disease severity, female sex, and native higher levels of tetrameric TTR concentration at the start of the treatment were the most relevant good predictors of response. 14

In contrast, in non-endemic regions, real-world studies have shown that tafamidis is not similarly effective. A recent German single center study 15 with a mean follow-up of 34 months demonstrated that 56% of the patients (36/64; most carriers of variants other than V30M) on tafamidis worsened at least one stage in the PND scale at the last follow-up. In an Italian study 16 ( *n* = 61; most carriers of variants other than V30M) that included mostly LO-ATTRV-PN patients, only 1/3 subjects appeared to benefit from tafamidis. Another study 17 with non-V30M LO-ATTRv-PN patients from France also observed that tafamidis was only effective in slowing disease progression in 1/3 patients after 3 years of follow-up. The response to tafamidis in ATTRv-PN patients appears to be different in endemic versus non-endemic regions, which is likely explained by different genotypes, time from symptom onset to diagnosis, and neuropathy severity at drug initiation.

In the present study, 75% of the patients responded to tafamidis. Only 10 patients presented progression of ther PND stage at the last follow-up. The median rate of NIS progression per year among the non-responders was of 11.3, which is similar to the expected rate of progression in untreated patients (11.7–14.3/year). 18 19 Given the difficult access to the very expensive gene therapy in Brazil, we advocate the use of this simple classification of responders and non-responders based on NIS progression of 7 or more points per year from tafamidis D1 after also considering the cardiac, renal, and nutritional aspects of the disease. In V30M ATTRv-PN patients who do not respond to tafamidis, we recommend switching to one of the gene-silencing drugs if available, but LT is still an option for nonresponding V30M patients.

We acknowledge several limitations to the current study. First, the small sample size may have influenced the results and precluded multivariate analyses. Second, half of the patients only had 1 follow-up visit at least 6 months after tafamidis initiation. Third, our center is located in an upper-middle income country, and not all patients underwent nerve conduction studies at the last follow-up visit given the real-world scenario. Furthermore, part of the patients were evaluated during the coronavirus disease 2019 (COVID-19) pandemic. Finally, the present study included only V30M ATTRv-PN patients; therefore, the efficacy of tafamidis in Brazilian non-V30M patients is still unknown.

In conclusion, Tafamidis proved to be a safe and effective disease-modifying therapy in ATTRv-PN, and it has the benefit of being easy to manage by patients and doctors. It has the most extensive accumulated experience of all ATTRv disease-modifying treatments, which is important, as patients may have to take the medication for decades. Tafamidis is also effective and has been approved for ATTRv-CM treatment. The gene-silencing drugs appear to be more potent, but more expensive, and with more adverse effects. For being safe, cheaper, and effective, tafamidis still has an important role in the treatment of ATTRv-PN, especially in very early stages of EO V30M patients.
